# Identification and Expression Patterns of Opsin Genes in *Phortica okadai*, the Only Confirmed Vector of *Thelazia callipaeda* in China

**DOI:** 10.3390/insects17101082

**Published:** 2026-10-19

**Authors:** Senqi Jia, Jiabing Wang, Liang Wang, Yikang Wang, Yang Luo, Yongliang Cao, Bo Luo, Lingjun Wang, Hui Liu, Juan Zhou

**Affiliations:** Department of Parasitology, School of Basic Medical Sciences, Zunyi Medical University, No. 6 Xuefu West Road, Xinpu New District, Zunyi 563000, Chinawanglingjun@zmu.edu.cn (L.W.)

**Keywords:** *Phortica okadai*, *Thelazia callipaeda*, opsin

## Abstract

In China, *Phortica okadai* is the only established vector for *Thelazia callipaeda* and transmits infective larvae to various animals feeding on ocular secretions. Insect opsins are key components of the visual system and play essential roles in host location. However, the opsin repertoire and phylogenetic relationships in *P. okadai* remain unknown. Here, we identified five opsin genes from adult *P. okadai* genomic data and profiled their expression across developmental stages and tissues by real-time polymerase chain reaction. The results showed that four opsin genes (*PoRh1*, *PoRh3*, *PoRh5*, and *PoRh6*) were predominantly expressed in adult compound eyes and peaked during the adult stage, suggesting important roles in vision-dependent behaviors of adult *P. okadai*. These findings provide a foundation for elucidating the visual mechanisms and host-seeking behavior of *P. okadai*.

## Simple Summary

In this study, five opsin genes were identified from the genome data of *Phortica okadai*, and their expression patterns were characterized by quantitative real-time polymerase chain reaction. These results offer a molecular basis for further investigations into the photoreception and visual ecology of *P. okadai*.

## 1. Introduction

*Phortica okadai* (Máca) (Diptera: Drosophilidae) feeds on ocular secretions and conjunctiva of animals [1] and is the only confirmed vector of *Thelazia callipaeda* (Spirurida: Thelaziidae) in China. It transmits infective larvae to a broad range of hosts, including wild and domestic animals and humans, causing thelaziasis. This infection is characterized by conjunctivitis, purulent discharge, epiphora, lacrimation, photophobia, chemosis, keratitis, and, in severe cases, corneal ulceration [2]. Understanding the host-seeking mechanism of *P. okadai* is essential for developing effective strategies to control thelaziasis.

Many insects possess elaborate visual systems that enable orientation and navigation, prey capture, habitat selection, and communication in diverse environments [3]. Adult insects perceive light primarily through the compound eyes and ocelli. The compound eyes consist of repeated functional units called ommatidia, each comprising a cornea and crystalline cone overlying a cluster of photoreceptor cells with a light-sensitive rhabdom. Within each ommatidium, variation in the number, morphology, and arrangement of photoreceptors shapes the capacity to detect color and polarized light [4]. Photoreceptors contain abundant rhodopsin, a visual pigment composed of an opsin covalently bound to a chromophore. Whereas most insect visual pigments share a common chromophore, 11-cis-retinal, their absorption maxima are primarily determined by the opsin [5,6,7]. Despite this central role in photoreception, the opsin repertoire of *P. okadai* remains unexplored.

Insect opsins are a class of transmembrane proteins with a size of typically 30–50 kDa and belong to the G protein-coupled receptor (GPCR) superfamily, consisting of seven transmembrane domains [8]. Opsins are divided into two major categories based on their direct involvement in visual imaging: visual opsins and non-visual opsins. Visual opsins are further categorized by spectral sensitivity: long-wavelength-sensitive opsin (LW opsin), blue light-sensitive opsin (BL opsin), and ultraviolet-sensitive opsin (UV opsin) [9].

*Nina E* (or *Rh1*) in *Drosophila melanogaster* is expressed only in R1-R6 photoreceptors and plays a major role in motion vision [10,11,12,13]. The *Rh2* opsin gene, which encodes a violet-light-sensitive pigment, was subsequently identified [14,15,16]. The *Rh3* and *Rh4* ultraviolet-sensitive opsin genes are expressed in R7 photoreceptor cells [17,18,19], while the *Rh5* blue-sensitive opsin gene and the *Rh6* long-wavelength-sensitive opsin gene are expressed in R8 photoreceptor cells [20]. Nevertheless, the number of opsins varies among insect species, owing to gene loss or duplication events. These differences highlight the adaptation of insects to their respective living environments [21]. 

Here, we focus on the opsin genes of *P. okadai* to lay the foundation for further studies on its host localization and eye-wiring mechanism.

## 2. Materials and Methods

### 2.1. Insect Rearing

The *P. okadai* used in the present study was collected from a field in Zunyi, Guizhou Province, China, and was reared in the laboratory. The insects were maintained in plastic containers measuring 27 cm in length, 20 cm in width, and 10 cm in height, supplied with naturally fermented fruits for three days at a temperature of 28 ± 1 °C, 75 ± 5% relative humidity, and a 16L:8D photoperiod.

### 2.2. Identification of Opsins

To identify opsin genes in *P. okadai*, full-length amino acid sequences of opsins from representative Diptera, Hymenoptera, and Lepidoptera were retrieved from GenBank and used as query sequences for local BLASTp searches (E-value cutoff < 1 × 10^−5^) against the *P. okadai* genomic assembly. Furthermore, a Hidden Markov Model (HMM) search was performed against the predicted protein dataset of *P. okadai* using the rhodopsin-like G-protein-coupled receptor HMM 3.0 profile (Pfam: PF00001). Redundant and incomplete sequences were manually filtered out. The raw genomic data have been deposited in the Genome Sequence Archive (GSA) at the China National Center for Bioinformation/Beijing Institute of Genomics, Chinese Academy of Sciences, under accession number CRA008976 (BioProject: PRJCA013354) [22]. The nucleotide sequences of the five *P. okadai* opsin genes (*PoRh1*, *PoRh3*, *PoRh5*, *PoRh6*, and *PoRh7*) characterized in this study are available in NCBI GenBank under accession numbers QB150445-QB150449.

### 2.3. Sequencing Analysis and Phylogenetic Tree Construction

The amino acid sequences of the opsin genes of *P. okadai* were compared using the DNAMAN v6.0 software (Lynnon Biosoft, Quebec, QC, Canada). The theoretical isoelectric points (pI), molecular weight (kDa), and instability indices were predicted with ExPASy ProtParam (https://web.expasy.org/protparam/, accessed on 17 October 2025). The functional sites of opsin were predicted by Prosite Scan (https://npsa-prabi.ibcp.fr/cgi-bin/npsa_automat.pl?page=/NPSA/npsa_proscan.html, accessed on 17 October 2025). 

For phylogenetic inference, opsin protein sequences from *P. okadai* and other representative insects were subjected to neighbor-joining (NJ) analyses. The resulting tree was visualized and annotated using iTOL (https://itol.embl.de/, accessed on 14 October 2026).

### 2.4. Quantification of Gene Expression Levels by qRT-PCR

Eggs (approximately 2 days old, 60 individuals per biological replicate), larvae (mixed instars, 20 individuals per biological replicate), pupae (approximately 5 days old, 10 individuals per biological replicate), and 3- and 8-day-old adults (10 individuals per biological replicate) of *P. okadai* were collected for developmental expression analysis. For tissue-specific expression detection, a total of 360 uniformly sized 8-day-old adults were used, with 120 adults per biological replicate (60 females and 60 males) dissected to collect five tissue types, including antennae, abdomen, compound eyes, thorax, and genitalia. All samples at each developmental stage and each tissue type were prepared with three independent biological replicates and three technical quantitative real-time polymerase chain reaction (qRT-PCR) replicates to reduce instrumental and operational variation.

Gene-specific primers were designed based on the open reading frames (ORFs) of the opsin genes using Primer Premier 6.0 software (Table 1). Total RNA was extracted using a total RNA extraction kit (Solarbio, Beijing, China), and cDNA was synthesized with a HiFiScript cDNA Synthesis kit (CoWin Biosciences, Beijing, China). qRT-PCR was performed in a 20 µL reaction volume using SYBR Premix Ex Taq II (Takara Biotechnology, Dalian, China) under the following conditions: 95 °C for 30 s, followed by 40 cycles of 95 °C for 5 s and 55 °C for 30 s. *β-actin* was used as the internal reference gene to normalize target gene expression [22].

### 2.5. Statistical Analysis

Relative expression levels were calculated using the 2^−ΔΔCT^ method and expressed as the means  ±  standard error (SE) [23]. Since these values followed a log-normal distribution and exhibited heterogeneous variance, the fold-change data were log_2_-transformed prior to statistical analysis to approximate a normal distribution and meet the assumptions for parametric testing. Data analysis and graphing were performed using GraphPad Prism 11. One-way analysis of variance (ANOVA) was used to compare the relative expression of opsin genes in *Phortica okadai* across different developmental stages. Two-way ANOVA was applied for comparisons across different tissues, followed by Tukey’s honestly significant difference (HSD) post hoc test for pairwise multiple comparisons. The significance threshold was set at *α* = 0.05. Adjusted *p* values and 95% confidence intervals of mean differences were reported. Compact letter display was used to indicate significant differences in figures.

## 3. Results

### 3.1. Opsin Identification and Sequence Analysis

**Figure 1:**
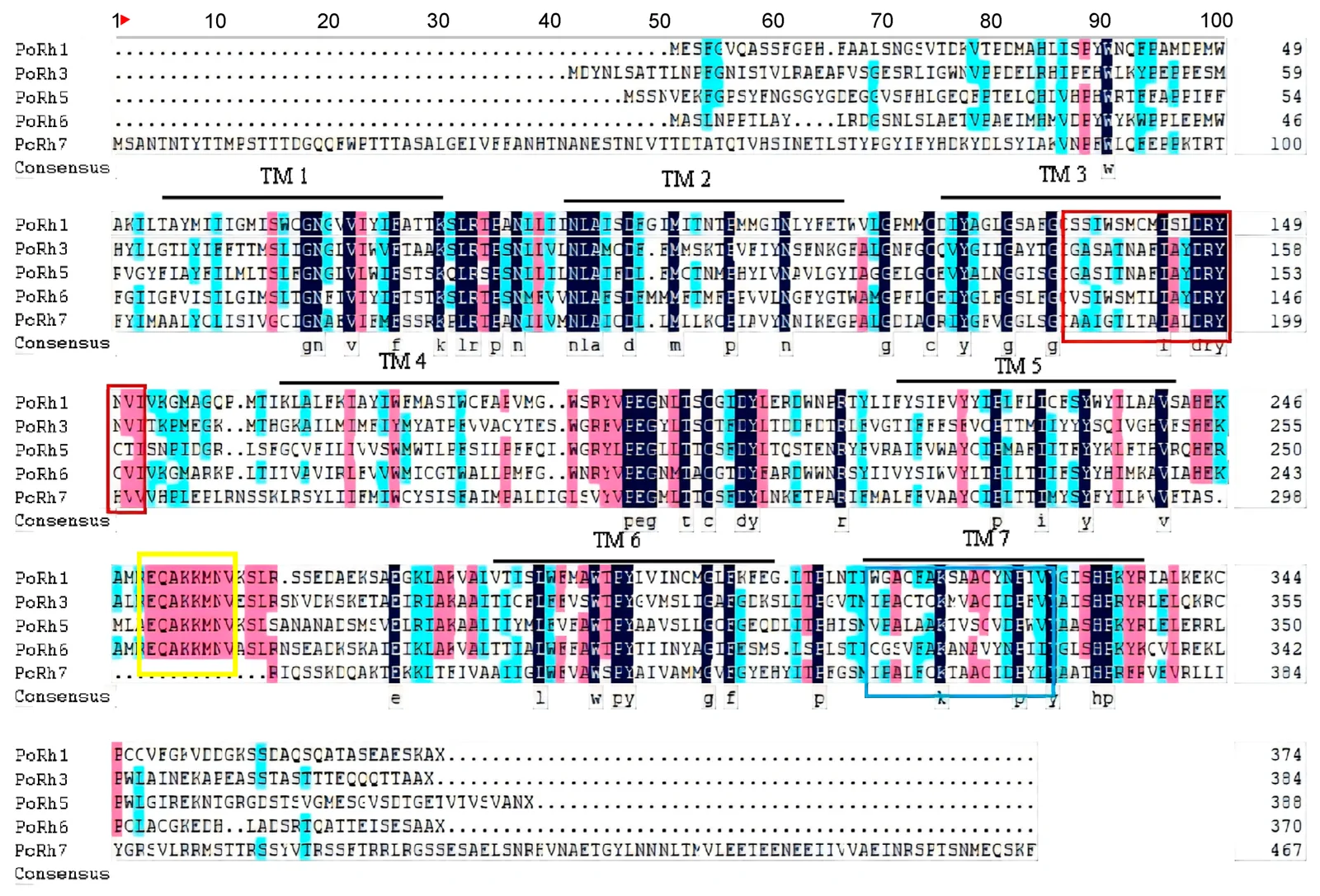
Sequence alignment of five opsins in *P. okadai*. TM1-TM7, transmembrane domains from 1 to 7. The G protein-coupled receptor family characteristics indicated are highlighted in red, the visual pigment (opsin) retinal binding site in blue, and the QAKKMNV motif (associated with G-protein binding) in yellow.

Based on the genomic data, we identified five opsin genes in *P. okadai*, named *PoRh1*, *PoRh3*, *PoRh5*, *PoRh6*, and *PoRh7*. The characteristics of predicted proteins for these five opsin genes are summarized in Table 2. These five opsins encoded 374, 384, 388, 370, and 468 amino acid residues, respectively. Multiple sequence alignments of these five opsins revealed that their functional sites contained the GPCR family signature, a visual pigment (opsin) retinal-binding site, and seven transmembrane (TM) domains (Figure 1). 

### 3.2. Phylogenetic Analysis of the Opsins

**Figure 2:**
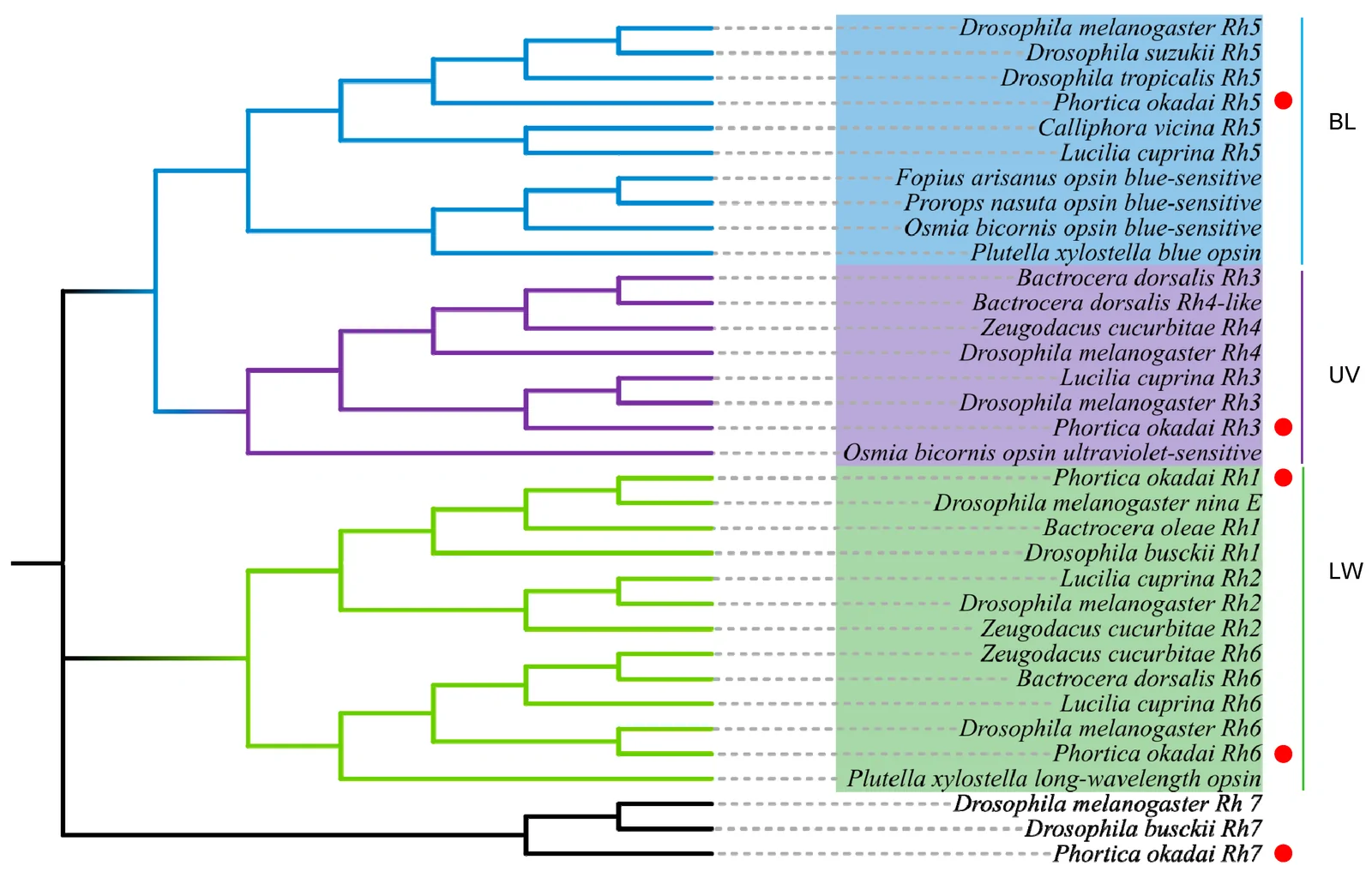
Neighbor-joining tree based on protein sequences of opsins from *P. okadai* and other insects. The other opsin genes correspond to Diptera, Hymenoptera, and Lepidoptera. Dots denote *P. okadai* opsin sequences.

To infer the evolutionary relationships among the five identified PoRh opsins of *P. okadai*, we performed phylogenetic reconstruction based on opsin amino acid sequences from diverse insect taxa covering Diptera, Lepidoptera, and Hymenoptera. NJ (Figure 2) tree showed that the long-wavelength-sensitive (LW), blue-sensitive (BL), ultraviolet-sensitive (UV), and Rh7 opsins of *P. okadai* clustered into four clades similar to those of other insects: PoRh1 and PoRh6 both belong to the LW opsin clade, PoRh3 to the UV opsin clade, and PoRh5 to the BL opsin clade. Each *P. okadai* opsin formed a sister relationship with dipteran orthologs, reflecting strong sequence conservation of these visual photoreceptor genes within Diptera.

### 3.3. Expression Levels of the Opsins During Different Developmental Stages

**Figure 3:**
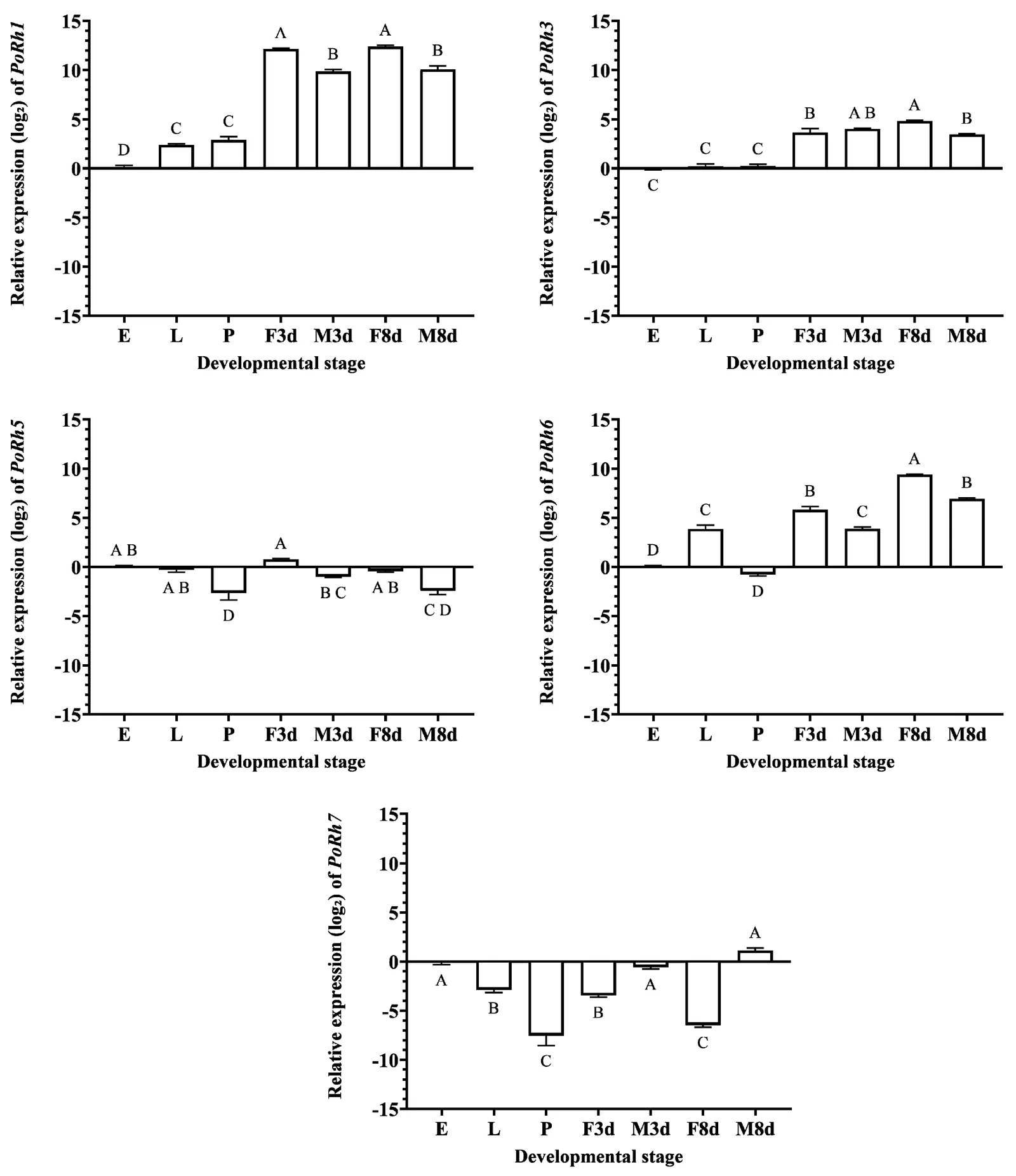
Relative expression levels of opsin genes in *P. okadai* at different developmental stages. E, eggs; L, larvae; P, pupae; F3d, 3-day-old female adults; M3d, 3−day−old male adults; F8d, 8−day−old female adults; M8d, 8−day−old male adults. Data are presented as log_2_−transformed relative expression levels; each value is the mean  ±  SE of three independent biological replicates. Different letters above the bars indicate significant differences between developmental stages based on a one−way ANOVA test followed by Tukey’s HSD post hoc test (α = 0.05).

Relative expression levels of five opsin genes (*PoRh1*, *PoRh3*, *PoRh5*, *PoRh6*, and *PoRh7*) were quantified across seven developmental stages of *P. okadai* (eggs, larvae, pupae, 3- and 8-day-old adults of both sexes) using qRT-PCR (Figure 3). Overall, the five genes exhibited distinct stage- and sex-specific expression patterns.

*PoRh1* and *PoRh3* exhibited similar expression patterns, with lower expression in eggs, larvae, and pupae and markedly higher levels in adults, and showed peak expression in 8-day-old females. For *PoRh1*, expression levels in 3- and 8-day-old females were significantly higher than those in all other developmental stages, while expression in 3- and 8-day-old males was significantly higher than that in eggs, larvae, and pupae. Eggs exhibited the lowest expression, which was significantly lower than that in all other stages. No significant differences were observed between 3- and 8-day-old females, between 3- and 8-day-old males, or between larvae and pupae. For *PoRh3*, expression levels in 3-day-old males did not differ significantly from those in 8-day-old females, 3-day-old females, or 8-day-old males. Expression in 3-day-old females and 8-day-old males was significantly lower than that in 8-day-old females, yet markedly higher than that in eggs, larvae, and pupae. Eggs, larvae, and pupae formed the lowest-expression group, with no significant differences detected among these three stages.

*PoRh5* exhibited relatively low and stable expression across all developmental stages, peaking in 3-day-old females. Eggs, larvae, and 8-day-old females displayed intermediate transcript levels; these groups were not significantly different from 3-day-old females and male adults but were significantly higher than pupae and 8-day-old males. Expression in 8-day-old males was not significantly different from pupae, which had the lowest expression.

*PoRh6* showed the highest expression in 8-day-old female adults, significantly higher than in 3-day-old females and 8-day-old males, and both groups had significantly higher transcript abundance than larvae and 3-day-old males. Larvae and 3-day-old males showed significantly higher levels than eggs and pupae. No significant differences were detected between 3-day-old females and 8-day-old males, or between 3-day-old females and larvae, or between eggs and pupae. 

*PoRh7* exhibited the highest transcript abundance in eggs, 3-day-old males, and 8-day-old males, which was significantly higher than that in larvae and 3-day-old females. These groups had significantly higher transcript levels than pupae and 8-day-old females. No significant differences were detected among eggs, 3-day-old males, and 8-day-old males, between larvae and 3-day-old females, or between pupae and 8-day-old females.

### 3.4. Expression Pattern of Opsin Genes in Different Tissues

**Figure 4:**
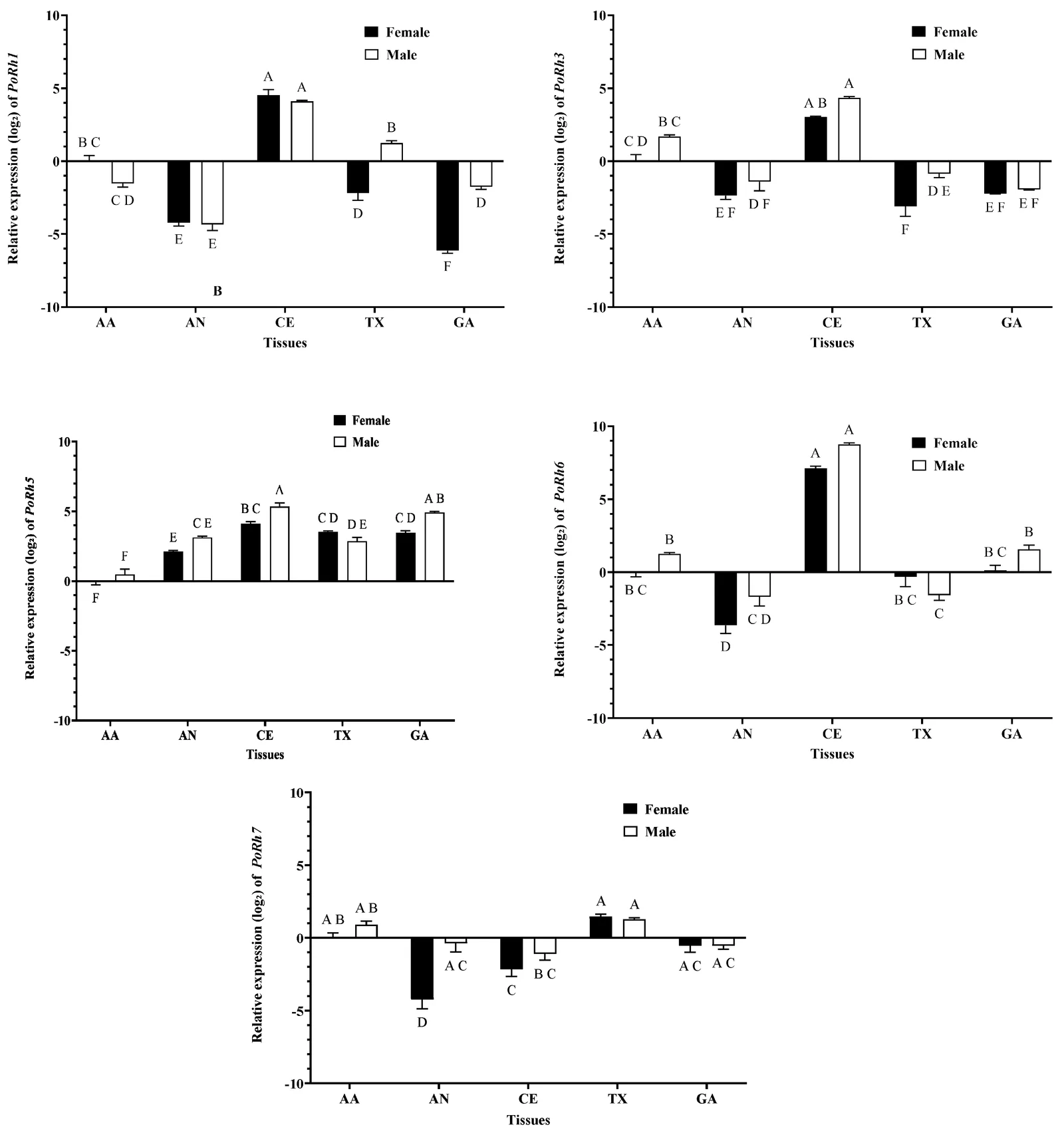
Relative expression levels of opsin genes in different tissues in *P. okadai* female and male adults. AA, antennae; AN, abdomen; CE, compound eyes; TX, thorax; GA, genitalia. Data are presented as log_2_−transformed relative expression levels. Each value is the mean  ±  SE of three independent biological replicates. Different letters above the bars indicate significant differences among tissue–sex groups, as determined by two−way ANOVA followed by Tukey’s HSD post hoc test (α = 0.05).

The relative expression levels of the five opsin genes (*PoRh1*, *PoRh3*, *PoRh5*, *PoRh6*, and *PoRh7*) were examined in the antennae (AA), abdomen (AN), compound eyes (CE), thorax (TX), and genitalia (GA) of adult female and male *P. okadai* using qRT-PCR (Figure 4).

For *PoRh1*, *PoRh3*, *PoRh5*, and *PoRh6*, compound eyes exhibited the highest expression in both sexes.

In females, antennae ranked second for *PoRh1* and *PoRh3* and were significantly higher than the thorax, abdomen, and genitalia. Significant differences were detected across the thorax, abdomen, and genitalia for *PoRh1*, whereas these three tissues showed comparable transcript abundance for *PoRh3*. *PoRh5* peaked in compound eyes, followed by the thorax, with antennae showing the lowest transcript abundance. For *PoRh6*, antennae, thorax, and genitalia had similar levels, all significantly higher than the abdomen. 

For *PoRh1* in males, compound eyes exhibited the highest expression, followed by the thorax, which was significantly higher than the antennae and genitalia. The abdomen showed the lowest expression, significantly lower than all other tissues. For *PoRh3*, compound eyes were followed by the antennae, which were significantly higher than the abdomen, thorax, and genitalia, with no significant differences detected among these three tissues. For *PoRh5*, compound eyes had the highest expression, followed by the thorax, which was significantly higher than the genitalia, antennae, and abdomen; the antennae had the lowest expression. In contrast, for *PoRh6*, expression was highest in compound eyes, followed by the antennae and genitalia, which were significantly higher than the thorax and abdomen; the abdomen showed the lowest expression but did not differ significantly from the thorax.

*PoRh7* exhibited the highest transcript abundance in the thorax, followed by the antennae in both sexes. In females, the genitalia showed the next highest expression, followed by the compound eyes and abdomen, with the abdomen showing the lowest expression. In males, the genitalia showed the next highest expression, followed by the abdomen and compound eyes, with the compound eyes showing the lowest expression.

For tissue expression profiles, significantly higher transcript levels in males than in females were observed for *PoRh1* in the thorax and genitalia, *PoRh3* in the thorax, *PoRh5* in compound eyes and genitalia, and *PoRh7* in the abdomen. No significant sex-based expression differences were detected in the other examined tissues.

## 4. Discussion

*P. okadai* is the primary confirmed vector of *T. callipaeda* in China, transmitting infective larvae during its feeding activities on host tear fluids [24]. However, the mechanisms underlying host recognition and localization by *P. okadai* remain unknown. Studies have demonstrated that vision predominates in short-range host location, and opsins are key photoreceptive proteins that mediate visual perception, circadian rhythms, and behavioral responses [25].

To elucidate the molecular basis of visual host recognition in *P. okadai*, we identified five opsin genes from genomic data and analyzed their expression patterns across different developmental stages and tissues. Multiple-sequence alignment confirmed that all identified PoRh proteins possess the canonical features of insect opsins, including seven transmembrane (7-TM) domains, GPCR family signatures, and opsin retinal-binding sites with a conserved lysine residue in TM7, which are typical characteristics of all opsins [26,27]. PoRh1, PoRh3, PoRh5, and PoRh6 contain the QAKKMNV motif, whereas PoRh7 lacks this motif entirely. The retention of QAKKMNV in PoRh1, PoRh3, PoRh5, and PoRh6 mirrors the pattern observed in conventional visual opsins of numerous insect taxa and underscores the evolutionary conservation of G protein-binding sites in image-forming rhodopsins [21,28]. By contrast, loss of the QAKKMNV motif in PoRh7 is consistent with observations in many insects: in *Drosophila melanogaster*, Rh7 does not couple to the conventional Gαq-dependent phototransduction cascade despite possessing the typical rhodopsin architecture and instead exhibits a preference for inhibitory-type G-protein subunits, a functional shift partly attributed to the absence of QAKKMNV [29]. We therefore speculate that PoRh7 may have similarly rewired its G-protein coupling specificity.

Molecular phylogenetic analyses identified five opsin protein in *P. okadai*, encoding long-wavelength (LW), ultraviolet (UV), and blue-sensitive (BL) opsins (Figure 2), which are consistent with those widely accepted in most insects. Notably, PoRh2 and PoRh4, which are present in *D. melanogaster*, were not detected [17]. Opsin gene loss has been documented in diverse insects; for example, blue-sensitive opsins have been independently lost in multiple Hemiptera (aphids, mirid bugs, planthoppers) and Coleoptera (*Tribolium castaneum*) [30,31,32], and subterranean beetles show parallel loss or pseudogenization of multiple opsin genes [33]. In *T. castaneum*, loss of the blue opsin is accompanied by expanded expression of LW opsin across all photoreceptors, suggesting functional compensation [34]. Similarly, the absence of PoRh2 and PoRh4 in *P. okadai* may be compensated by the retained LW (PoRh1, PoRh6) and UV (PoRh3) opsins, though this hypothesis requires experimental validation. 

Overall, *PoRh1*, *PoRh3* and *PoRh6* exhibited higher expression in adult compound eyes than in other developmental stages or tissues, suggesting important roles in the visual system of *P. okadai* that may be associated with visually guided behaviors such as flight, host-seeking, and oviposition-site selection (Figure 3 and Figure 4). 

The 3-day-old and 8-day-old adults correspond to the stages prior to and following sexual maturation, respectively. The results suggest that the marked increase in transcript abundance of *PoRh3* and *PoRh6* in females from 3-day-old to 8-day-old adults may be involved in visual host location for oviposition. A prior study demonstrated that knockdown of *Rh6* in *Bactrocera minax* substantially reduces oviposition on green unripe citrus fruits [35]; nevertheless, whether *Rh3* participates in oviposition-site selection awaits further experimental validation. The highest expression of *PoRh1* in female compound eyes (Figure S2) is likely because Rh1 is responsible for motion detection and image formation [36]. 

For *PoRh5*, no marked differences in expression were observed among eggs, larvae, and adults. In terms of tissue distribution, no significant differences were detected among the compound eye, thorax, and genitalia in females, or between the compound eye and genitalia in males, suggesting that in addition to its canonical visual function, *PoRh5* may participate in other physiological processes, such as temperature preference or hearing [37]. 

*PoRh7* was predominantly expressed in eggs, larvae, and pupae (Figure S1), and its expression was mainly localized to the thorax. This thoracic enrichment is consistent with the non-visual role of *Rh7*. In *D. melanogaster*, *Rh7* is expressed in dorsal clock neurons, including *DN1p*, *DN1a*, and *DN2* neurons. *Rh7* mutants exhibit decreased night activity and fail to adjust their behavior to darkness, while daily activity levels remain similar to controls, suggesting that these effects reflect altered circadian regulation rather than general locomotor impairment [38]. Given that thoracic ganglia are primary motor centers controlling locomotion, we predicted that *PoRh7* in *P. okadai* may be involved in light-dependent modulation of activity patterns, thereby explaining its thoracic enrichment.

Insect opsins function as polymodal sensory receptors rather than exclusively as light sensors, which may explain their expression in non-visual tissues. In *Aedes aegypti*, *op1* and *op2* mRNAs are present in neurons in the most distal region of the antenna, where the corresponding opsins function as heat sensors [39]. In *Nilaparvata lugens*, the long-wavelength opsin gene participates in the regulation of cuticle melanization [40]. These findings collectively support the non-visual roles of opsins in non-visual tissues.

## 5. Conclusions

In conclusion, we identified five opsin genes from the genomic data of *P. okadai*. Expression profiling showed that four *PoRh* genes (excluding *PoRh7*) were highly expressed in adults and compound eyes. By contrast, *PoRh7* exhibited low expression in compound eyes yet was enriched in the thorax. Our findings suggest potential involvement of these opsins in adult-specific visual physiological processes. However, since no gene-functional or behavioral assays were performed in this study, the precise biological functions of these opsins still require validation in future experimental work. This study improves our knowledge of the visual system in *P. okadai* and may provide insights for future vector control strategies targeting light-dependent behaviors.

## Figures and Tables

**Table 1:** Primers used in this study.

Gene Name	Primers (5′-3′)	Nucleotide Position
*PoRh1*	Forward: CTGAGGATGCTGAGAAGAG	788–806
	Reverse: GCTGATGCCGTAGACAAT	979–996
*PoRh3*	Forward: GGCACGCTCTACATATTCT	190–208
	Reverse: ATTGGCTTGGTGATGACAT	476–494
*PoRh5*	Forward: TGATTGTTGTGTCGTGGAT	515–533
	Reverse: ATGCGGCGTTATTAAGTCT	927–945
*Po* *Rh6*	Forward: TCTGTTCGTTGTATGGATGA	498–517
	Reverse: ACCGTAGATGATTGGATTGT	965–984
*Po* *Rh* *7*	Forward: AAGCACGAATGATGTCACA	988–1007
	Reverse: ACGCGAAATCTGGGATGTGT	1133–1114
*Po* *β-actin*	Forward: TCCGCACTTTGAAACTGAC	635–653
	Reverse: CACGAGATGTTAGTGGAGCA	810–829

**Table 2:** Sequence features of five opsin genes and their encoded amino acids.

Name	ORF (bp)	Protein Length (aa)	Molecular Weight (kDa)	Theoretical pI	Instability Indices (II)
*PoRh1*	1125	374	41.76	8.32	21.96
*PoRh3*	1155	384	43.14	7.96	44.59
*PoRh5*	1167	388	43.51	6.75	36.99
*PoRh6*	1113	370	41.99	8.77	32.14
*PoRh7*	1407	468	52.56	8.22	47.94

## Data Availability

All data is provided in the manuscript.

## Supplementary Materials

The following supporting information can be downloaded at https://www.mdpi.com/article/10.3390/insects17101082/s1.
