# Supplementary material for: Identification and Expression Patterns of Opsin Genes in Phortica okadai, the Only Confirmed Vector of Thelazia callipaeda in China
Source: Insects. 2026 Oct 19;17(10):1082. doi: 10.3390/insects17101082 (PMC13704940; doi:10.3390/insects17101082)
Supplement: Supplementary file 1 [file insects-17-01082-s001.zip › insects-4605943-supplementary.pdf]

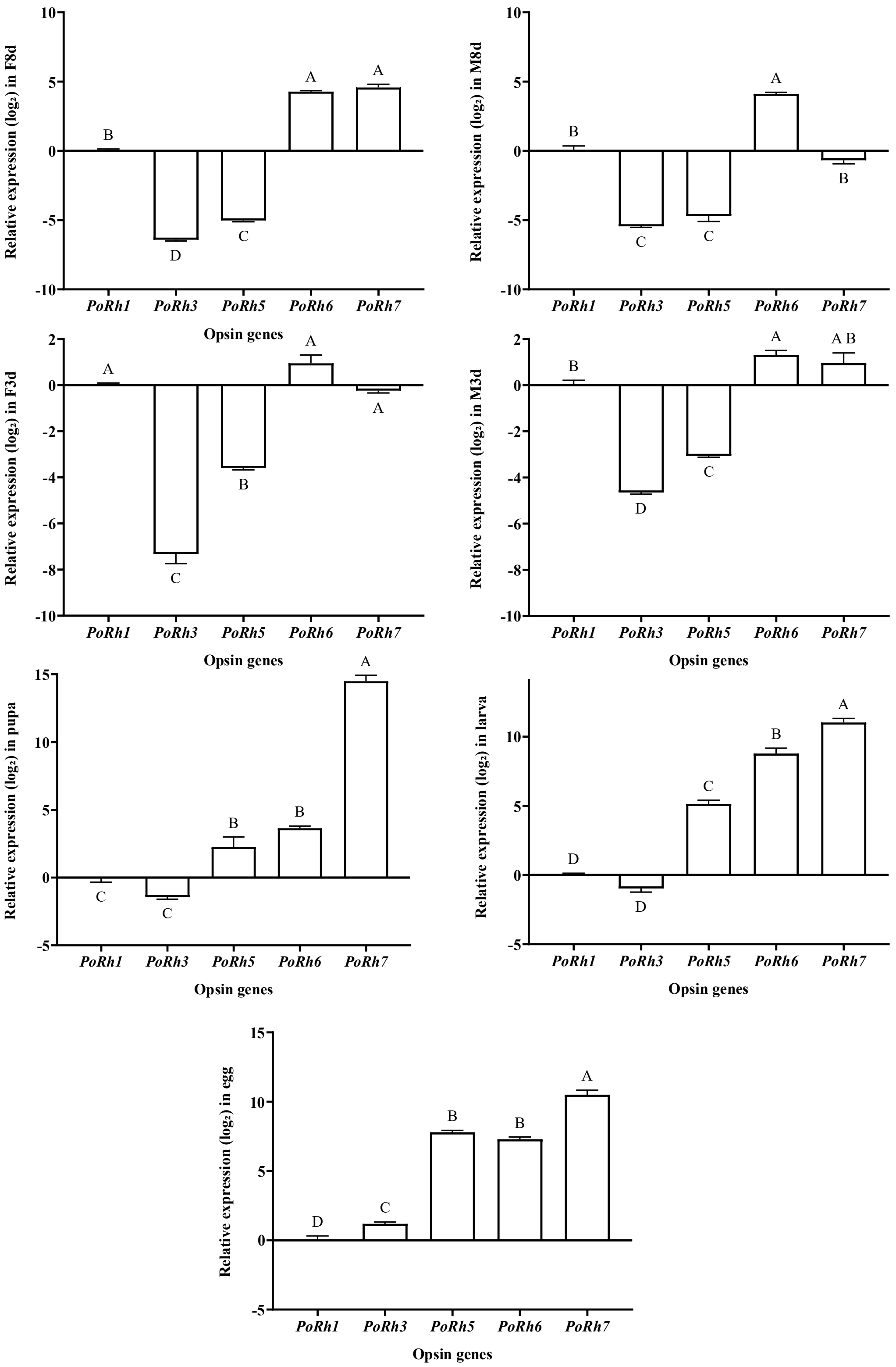

Figure S1. Relative expression levels of opsin genes in *P. okadai* at different developmental stages

Data are presented as log_2-transformed relative expression levels, each value is the mean ± SE of three collections. Different letters above the bars indicate significant differences between developmental stages based on a one- way ANOVA test (Tukey's HSD test, α=0.05).

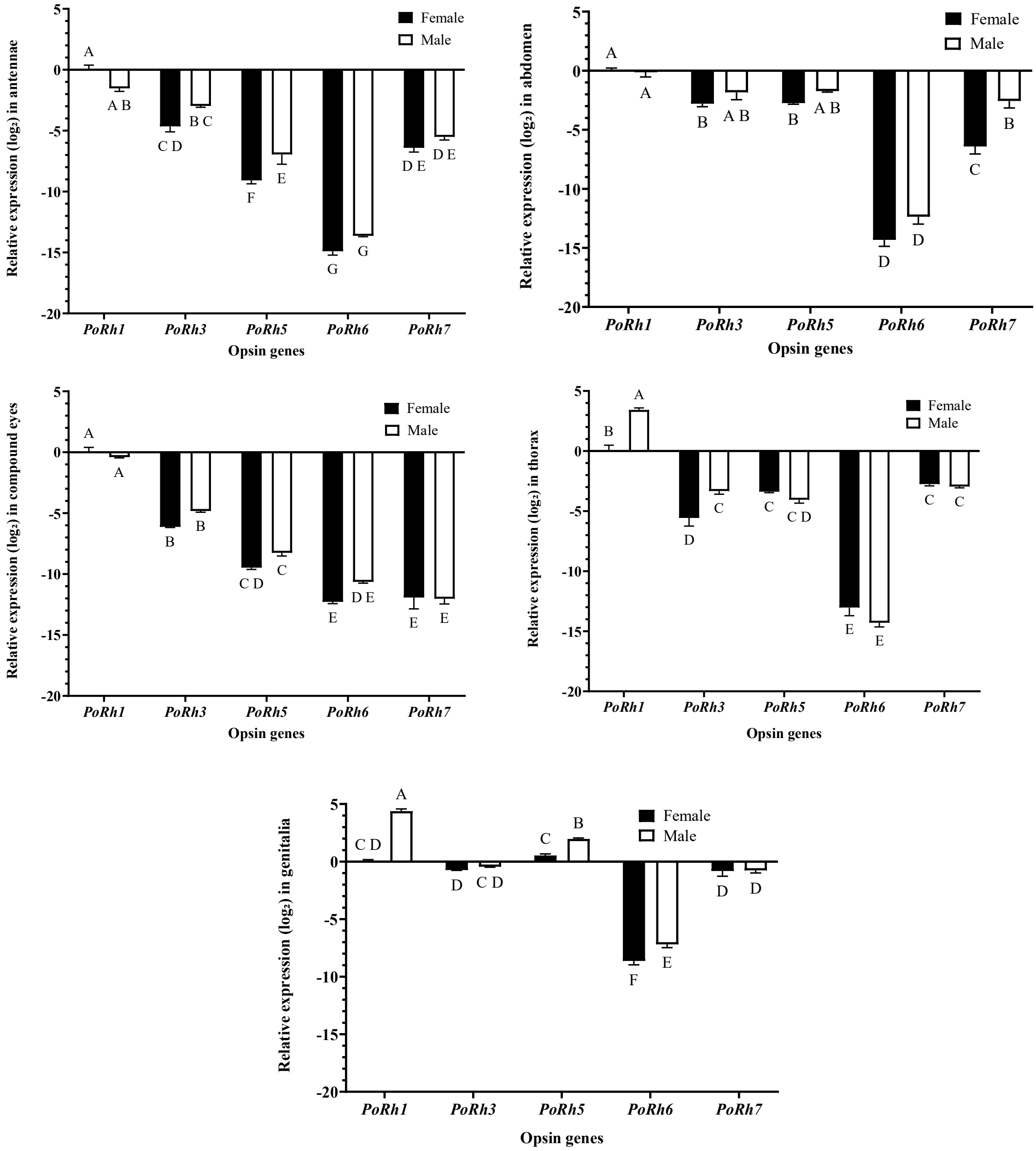

Figure S2. Relative expression levels of opsin genes in different tissues in *P. okadai* female and male adults.

Data are presented as log_2-transformed relative expression levels, each value is the mean ± SE of three collections. Different letters above the bars indicate significant differences between developmental stages based on a two-way ANOVA test (Tukey's HSD test, α=0.05).
